# Temporal Trends and Patient Stratification in Lung Cancer: A Comprehensive Clustering Analysis from Timis County, Romania

**DOI:** 10.3390/cancers17142305

**Published:** 2025-07-10

**Authors:** Versavia Maria Ancusa, Ana Adriana Trusculescu, Amalia Constantinescu, Alexandra Burducescu, Ovidiu Fira-Mladinescu, Diana Lumita Manolescu, Daniel Traila, Norbert Wellmann, Cristian Iulian Oancea

**Affiliations:** 1 Department of Computer and Information Technology, Automation and Computers Faculty, “Politehnica” University of Timisoara, Vasile Pârvan Blvd, no. 2, 300223 Timisoara, Romania; versavia.ancusa@upt.ro; 2 Center for Research and Innovation in Personalized Medicine of Respiratory Diseases (CRIPMRD), ‘Victor Babes’ University of Medicine and Pharmacy, Eftimie Murgu Square no. 2, 300041 Timisoara, Romania; ana.trusculescu@umft.ro (A.A.T.); amalia.constantinescu@umft.ro (A.C.); traila.daniel@umft.ro (D.T.); norbert.wellmann@umft.ro (N.W.); oancea@umft.ro (C.I.O.); 3 Pulmonology University Clinic, Clinical Hospital of Infectious Diseases and Pneumophysiology, Dr. Victor Babeș Timișoara, Gheorghe Adam Street, no. 13, 300310 Timisoara, Romania; alexandra.burducescu.umfvbt@gmail.com; 4 Doctoral School, “Victor Babes” University of Medicine and Pharmacy Timisoara, Eftimie Murgu Square 2, 300041 Timisoara, Romania; 5 Radiology and Medical Imaging University Clinic, Clinical Hospital of Infectious Diseases and Pneumophysiology, Dr. Victor Babeș Timișoara, Gheorghe Adam Street, no. 13, 300310 Timisoara, Romania

**Keywords:** lung cancer, machine learning, patient clustering, epidemiology, personalized medicine, temporal trends, Romania

## Abstract

**Background/Objectives:** Lung cancer remains a major cause of cancer-related mortality, with regional differences in incidence and patient characteristics. This study aimed to verify and quantify a perceived dramatic increase in lung cancer cases at a Romanian center, identify distinct patient phenotypes using unsupervised machine learning, and characterize contributing factors, including demographic shifts, changes in the healthcare system, and geographic patterns. **Methods:** A comprehensive retrospective analysis of 4206 lung cancer patients admitted between 2013 and 2024 was conducted, with detailed molecular characterization of 398 patients from 2023 to 2024. Temporal trends were analyzed using statistical methods, while k-means clustering on 761 clinical features identified patient phenotypes. The geographic distribution, smoking patterns, respiratory comorbidities, and demographic factors were systematically characterized across the identified clusters. **Results:** We confirmed an 80.5% increase in lung cancer admissions between pre-pandemic (2013–2020) and post-pandemic (2022–2024) periods, exceeding the 51.1% increase in total hospital admissions and aligning with national Romanian trends. Five distinct patient clusters emerged: elderly never-smokers (28.9%) with the highest metastatic rates (44.3%), heavy-smoking males (27.4%), active smokers with comprehensive molecular testing (31.7%), young mixed-gender cohort (7.3%) with balanced demographics, and extreme heavy smokers (4.8%) concentrated in rural areas (52.6%) with severe comorbidity burden. Clusters demonstrated significant differences in age (*p* < 0.001), smoking intensity (*p* < 0.001), geographic distribution (*p* < 0.001), as well as molecular characteristics. COPD prevalence was exceptionally high (44.8–78.9%) across clusters, while COVID-19 history remained low (3.4–8.3%), suggesting a limited direct association between the pandemic and cancer. **Conclusions:** This study presents the first comprehensive machine learning-based stratification of lung cancer patients in Romania, confirming genuine epidemiological increases beyond healthcare system artifacts. The identification of five clinically meaningful phenotypes—particularly rural extreme smokers and age-stratified never-smokers—demonstrates the value of unsupervised clustering for regional healthcare planning. These findings establish frameworks for targeted screening programs, personalized treatment approaches, and resource allocation strategies tailored to specific high-risk populations while highlighting the potential of artificial intelligence in identifying actionable clinical patterns for the implementation of precision medicine.

## Simple Summary

Lung cancer is one of the leading causes of cancer deaths worldwide, and its patterns can vary greatly depending on local factors. Our hospital in Timisoara, Romania, observed a dramatic 80% increase in lung cancer patients after the COVID-19 pandemic, prompting us to investigate whether this represented a true epidemiological shift. Using advanced machine learning techniques on detailed medical records from 4206 patients over a 12-year period, we confirmed this increase and identified five distinct patient groups with unique characteristics. These included elderly never-smokers with high rates of advanced disease, extremely heavy smokers concentrated in rural areas with severe respiratory complications, and young patients with balanced gender distribution. Our analysis revealed that the post-pandemic increase reflected genuine epidemiological changes beyond healthcare system factors, supported by regional demographic aging and enhanced diagnostic practices. This research demonstrates how artificial intelligence can uncover hidden patient patterns to inform personalized medicine approaches and guide targeted prevention strategies for specific high-risk populations in our region.

## 1. Introduction

### 1.1. Research Gap

Lung cancer remains one of the leading causes of cancer-related mortality worldwide, with significant regional variations in incidence, presentation, and outcomes. While global epidemiological patterns have been well-documented, localized clustering of lung cancer cases with distinct clinical and pathological features can provide crucial insights for regional healthcare planning and targeted interventions.

### 1.2. Background on Lung Cancer Epidemiology and Heterogeneity

Globally, about 2.5 million new lung cancer cases and 1.8 million deaths were reported in 2022, with incidence and mortality rates varying widely by region [1]. In Europe, lung cancer rates are higher in Eastern countries and consistently greater in men than women, reflecting differences in smoking prevalence and other risk factors [2].

Romania registered 7512 new lung cancer cases in 2019 (38.8 per 100,000 inhabitants), showing a downward trend from 41.2 per 100,000 in 2010 [3]. However, 2022 data indicate 11,857 new cases (8819 males, 3038 females) [4], contradicting this trend. The matter is further complicated by the lack of a national cancer registry and the fragmentation of cancer incidence reporting, resulting in incomplete data [4]. In Timiș County, bronchopulmonary cancers accounted for 27.09% of all new cancer cases in 2022, with rural incidence exceeding urban rates (29.87% vs. 25.16%), and demonstrated a gender disparity with a male-to-female ratio of approximately 2.2:1 (35.46% of new male cancers compared to 16.49% in females) [5].

### 1.3. The Rationale for Patient Clustering (Improved Treatment Selection, Prognostic Value)

A better understanding of disease heterogeneity, which enables more precise patient stratification, can improve treatment decisions and prognostic assessments by revealing patterns and relationships that may not be apparent through traditional analyses. Previous unsupervised analysis techniques have demonstrated that patient clustering in lung cancer enables the identification of distinct patient subgroups based on clinical and molecular data without prior assumptions, leading to an improved understanding of cancer heterogeneity, better prognostic stratification, and the possibility of developing personalized medicine strategies [6].

### 1.4. Literature Review of Previous Clustering Attempts in Lung Cancer

Several machine learning studies conducted in the lung cancer field have shown that this technique may become a powerful tool in cancer research for uncovering novel patient subgroups based on complex clinical, molecular, imaging, and multi-omics data [7]. Enabling the integration and analysis of complex data facilitates a more accurate classification of lung cancer subtypes than other traditional methods [7].

### 1.5. Hypothesis and Objectives

Recent clinical observations at our center have suggested a subjective increase in lung cancer diagnoses over the past few years, raising important questions about potential regional shifts in disease patterns. This perceived change warrants rigorous investigation to determine whether it represents a true epidemiological trend or an artifact of improved detection methods or referral patterns.

If the change is not subjective, then its potential drivers demand careful analysis. The COVID-19 pandemic has introduced multiple factors that could influence lung cancer presentation, including delayed diagnoses due to healthcare avoidance, potential lung tissue damage, increasing susceptibility, and altered healthcare-seeking behaviors. Additionally, the complex interplay between pre-existing respiratory conditions, such as Chronic Obstructive Pulmonary Disease (COPD) and asthma, with lung cancer development remains inadequately characterized in our specific patient population. Environmental factors, particularly regional air pollution patterns, represent another potential contributor to the changing presentation of lung cancer. Recent studies have demonstrated associations between particulate matter exposure and lung cancer risk, even among never-smokers, but the relevance of these findings to our specific geographic context remains unclear.

Despite the extensive literature on lung cancer patient classification, few studies have employed unsupervised clustering approaches to identify naturally occurring patient subgroups within geographically defined populations. This gap is particularly pronounced for our region, where unique demographic, environmental, and healthcare access factors may create distinctive patient clusters that differ from those described in broader populations.

This study aims to address these knowledge gaps by

1. Quantifying temporal trends in lung cancer cases to verify perceived increases in incidence;
2. Identifying distinct lung cancer patient clusters within our regional population using k-means clustering of comprehensive clinical, pathological, and geographical data;
3. Investigating potential contributing factors to any observed increases, with a specific focus on the following:
  - COVID-19 history and sequelae;
  - Pre-existing respiratory conditions (COPD, asthma);
  - Geographical correlation;
4. Characterizing region-specific lung cancer presentations to inform locally tailored screening and treatment approaches.

By addressing these questions, we aim to provide actionable insights for regional healthcare planning, potentially identifying high-risk subpopulations that may benefit from targeted screening or preventive interventions.

## 2. Materials and Methods

### 2.1. Study Design and Patient Selection

This retrospective cohort study analyzed data from 4206 first primary lung cancer patients, with 5145 total visits, admitted to the “Victor Babes” University Hospital in Timisoara, Romania, between 1 January 2013 and 31 December 2024. Lung cancer diagnoses were classified according to the World Health Organization (WHO) classification of thoracic tumors [8]. Medical data was extracted from the healthcare facility’s digital patient record system.

Patients were eligible if they fulfilled the following:

- Were aged 18 years or older;
- Medical records included at least one diagnosis code;
- Had a confirmed diagnosis of primary lung cancer (ICD-10 codes C34.0-C34.9, D38.1, D38.6);
- Resided in Romania;
- Were admitted as inpatients (hospitalization ≥ 12 h) with a confirmed lung cancer diagnosis during the hospitalization period;
- Provided written informed consent for research purposes.

Exclusion criteria comprised the following:

- Age below 18 years;
- Unknown or non-Romanian home address;
- Diagnosis of secondary lung malignancy, indicating a metastatic disease from other primary sites;
- Death Certificate-Only cases and post-mortem discoveries of lung cancer diagnosis;
- Absence of informed consent;
- Outpatient status (due to inconsistent data availability).

This study employed a two-cohort approach based on the availability and quality of the pathology data. Patients diagnosed between 1 January 2013 and 31 December 2022 constitute the first cohort, which provides an epidemiological context for the overall lung cancer population. However, comprehensive pathology data for this period was limited because our digital patient record system did not store detailed pathology information prior to 2022. Additionally, pathology analyses were performed at multiple external centers using different formats and terminology, with results often sent directly to oncologists at other healthcare institutions, preventing systematic consolidation in our hospital’s database.

Beginning 1 January 2023, we established a second cohort by systematically collecting a private database containing comprehensive pathology data, including biopsy results, immunohistochemistry, and molecular findings. This post-2023 cohort enables detailed molecular characterization through manual curation of multi-source pathology data. To ensure analytical validity, we excluded inconsistent findings (29.1% of post-2023 cases) despite the challenges posed by decentralized laboratory workflows.

This bifurcated approach addresses critical data quality differences while maintaining scientific rigor: the pre-2023 cohort provides population-level epidemiological context with available clinical data, while the post-2023 cohort supports in-depth molecular analyses through systematically collected, high-quality pathology data.

Death certificate-only (DCO) cases were excluded from both cohorts due to insufficient clinical and pathological data.

Both cohorts were further refined using detailed smoking status information, including current/former/never smoking classifications and pack-year history, aligned with SNOMED CT clinical terminology [9].

Smoking-history data were manually reviewed and standardized by three independent clinical investigators (A.C., N.W., and D.T.) using the standardized smoking-assessment protocol [10,11,12,13]. Discrepancies in smoking-status classification (never/former/active) and pack-year calculations from different laboratories were resolved through consensus review. Pack-year exposure was calculated using the standard formula: (cigarettes per day ÷ 20) × years smoked. Inter-rater agreement for smoking status classification was assessed using Cohen’s kappa coefficient (κ = 0.8). All investigators underwent training on the standardization protocol prior to data review.

All patients from 2013 onward provided written informed consent for research purposes per institutional protocol.

The study protocol received ethical approval from both the university ethics committee (38/24.11.2023) and the hospital ethics committee (10535/13.11.2023) and 4904/30.05.2025.

### 2.2. Clinical Data Collection

#### 2.2.1. Demographic and Clinical Variables

Demographic parameters (age in completed years, binary sex categorization) and comorbidity profiles were extracted from the hospital’s electronic medical records system.

For the pathology subset cohort, the smoking history (status classification-never, former, current, and quantified pack-year exposure) was manually standardized by clinical investigators following a validated protocol.

Data collection was split into two periods:

→ 2013–2022: Data extracted from electronic records: pathology and molecular data were limited due to decentralized reporting and were used primarily for epidemiological context.
→ 2023–2024: After the COVID-19 pandemic, the increased use and improved documentation of CT imaging and lung biopsy, combined with manual curation from multiple sources, resulted in a more comprehensive and accurate collection of pathology, molecular, and clinical data. This enabled detailed molecular and cluster analyses.

#### 2.2.2. Pathological Assessment Methodology

Histopathological, immunohistochemical, and molecular data were manually curated and standardized by specialist physicians following receipt of external laboratory reports, ensuring consistency in classification nomenclature and biomarker interpretation across multiple referral laboratories. All pathological variables were subsequently coded according to the WHO Classification of Thoracic Tumors to facilitate statistical analysis [8].

Biopsy Procedures:

Lung tissue specimens were obtained using two complementary approaches:

- Standard bronchoscopic biopsy for centrally located lesions;
- CT-guided transthoracic biopsy when bronchoscopy failed (n = 89/398, 22.36%) or for peripheral tumors inaccessible via endoscopic routes (n = 127/398, 31.91%).

Anaplastic lymphoma kinase (ALK) and programmed death-ligand 1 (PD-L1) status were assessed by immunohistochemistry (IHC), while molecular methods evaluated epidermal growth factor receptor (EGFR) mutations; in selected cases with limited tissue, EGFR mutation-specific IHC was also employed, with negative or equivocal results confirmed by molecular testing [14].

#### 2.2.3. Geocoding Methods

The retrospective nature of this longitudinal study presented methodological challenges in assessing environmental exposures. Despite acknowledged limitations in capturing mobility patterns, patient residential addresses at the moment of hospitalization were employed as a pragmatic proxy for exposure assessment. A raw geographic distribution analysis revealed a predictable predominance of cases in Timisoara, reflecting its disproportionate demographic concentration (33% of the county population within 1.5% of the territorial area) [15,16].

A limitation of our geographic analysis is that the classification system was based on 2021 population data and did not account for demographic changes that may have occurred during the 12-year study period.

To facilitate machine learning applications, we implemented a four-tier geospatial classification system comprising the following classes: (1) Timisoara Metropolitan Core; (2) Timis County Sub-Urban Areas, with administrative units having more than 10,000 residents representing secondary population centers; (3) Timis County Rural Areas, encompassing administrative units with <10,000 residents; and (4) Extra-jurisdictional Cases originating outside Timis County, representing potential referral pattern outliers beyond the institution’s primary catchment area. This last category might also represent the migration pattern specific to the higher economic status in our county, but without further data, we cannot better characterize it. For the whole list of administrative units included in each category, please refer to Appendix A.

Environmental exposure assessment represents a significant limitation in our retrospective analysis. While our center has previously documented air pollution impacts on respiratory disease presentation in this region, comprehensive environmental data integration was not feasible for this study [17,18]. Timiș County exhibits elevated natural radon concentrations, according to EU digital atlas data [19]; however, the measurement density remains insufficient for robust patient-level exposure correlation, with only 180 houses monitored across the 8697 km^2^ representing Timiș County. Current regional radon mapping relies primarily on mathematical extrapolation to 10 × 10 km^2^ grids rather than comprehensive field measurements, which limits precise exposure quantification for individual patients [18].

We selected all unique identifiers and, for each identifier, determined its corresponding geolocation classes. For example, if an individual had both Class 1 and Class 2 addresses registered in the entire database, both classes were considered in machine learning. The rationale is that even though the address might change and the onset of lung cancer is found when, for example, the patient lives in a Class 2 area, they might have lived earlier in a different Class area, being exposed to that particular environment, which might have influenced the pathology. By using this classification schema, population density and mobility confounders were controlled. Also, this manual hierarchical clustering type pre-analysis reduced the number of parameters for clustering.

### 2.3. Data Analysis

We consolidated two distinct institutional databases for comprehensive analysis: a radiological database and a general clinical database. Due to the asymmetric distribution of radiological parameters between sources, these variables were excluded to maintain cohort integrity. Similarly, anthropometric measurements (weight and height) were omitted due to substantial missing data (n = 171) that prevented reliable imputation.

Next, all missing values were coded as zero, following established practices for categorical absence indicators. Ordinal categorical variables underwent indicator encoding; specifically, smoking status was restructured from its original ordinal scale (0 = never, 1 = active, 2 = former) into three binary variables representing each state. Diagnostic classifications were converted into a presence/absence binary matrix using ICD-10 codes, generating a high-dimensional sparse matrix (761 features × 398 patients).

**Figure A2:**
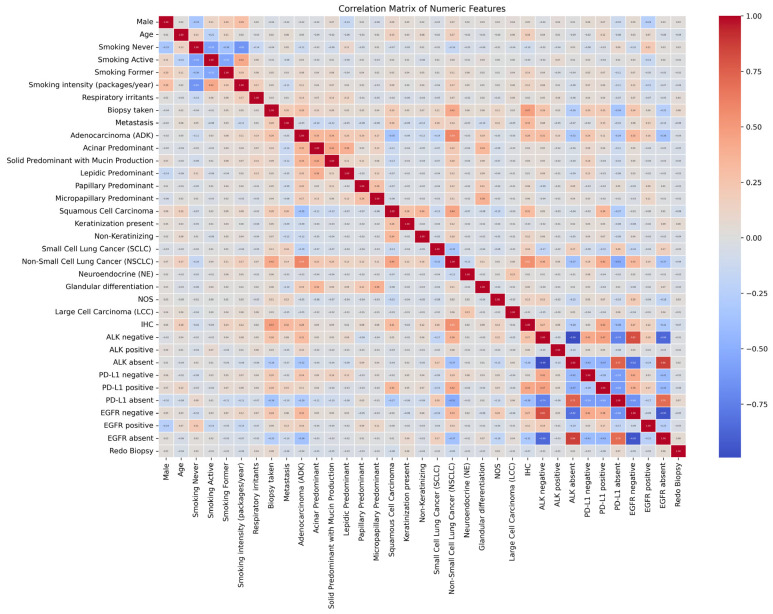
Heatmap of main features.

The intercorrelations among numerical variables were computed using Pearson correlation coefficients and visualized via a hierarchical clustered heatmap to identify redundant features and potential confounding relationships (Appendix B, Figure A2). Although there are some (expected) correlations between immunohistochemistry markers, we decided to keep them all.

Prior to model training, data preprocessing included feature scaling to ensure that all features contributed equally to the model. Specifically, a Standard Scaler was applied, transforming each feature such that its mean was 0 and its variance was 1.

**Figure A3:**
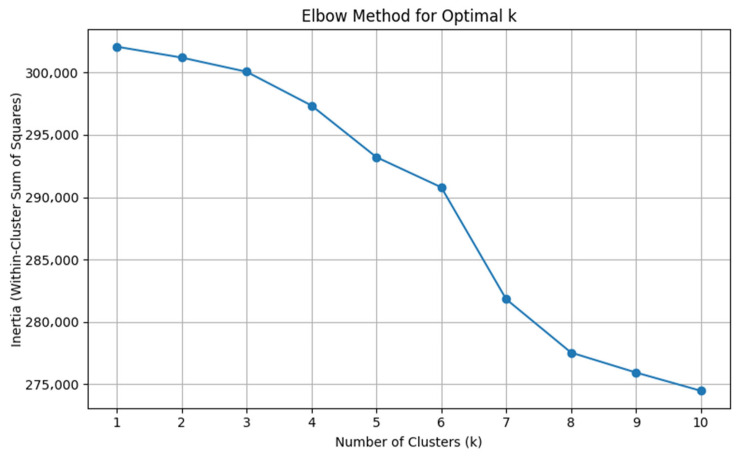
Elbow method.

**Figure A4:**
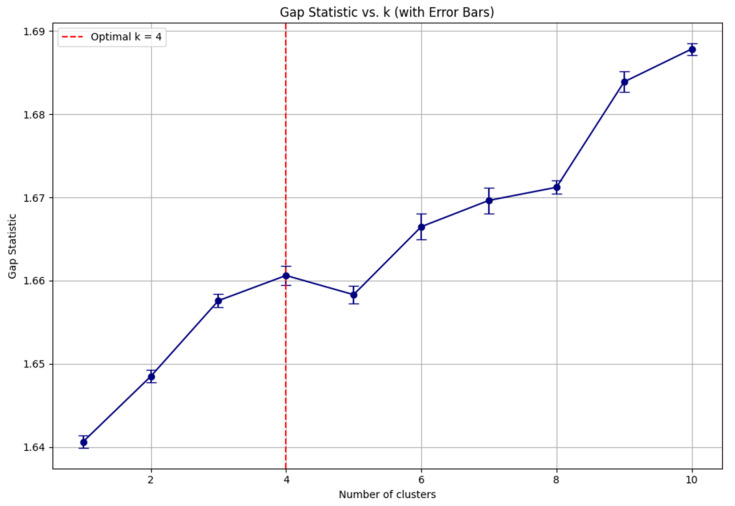
Gap statistics with standard deviation.

We employed multiple complementary methodologies for optimal cluster number determination:

- An elbow method analysis assessed the within-cluster sum of squares decline across sequential k values (Appendix B, Figure A3);
- Gap statistics with standard deviation quantified the clustering performance relative to null reference distributions (Appendix B, Figure A4);
- Comparative evaluation of internal validation metrics across candidate solutions (k ∈ {4,5,6}) using the Silhouette Score, Calinski–Harabasz Index, and Davies–Bouldin Score (Appendix B, Table A2)

**Figure A5:**
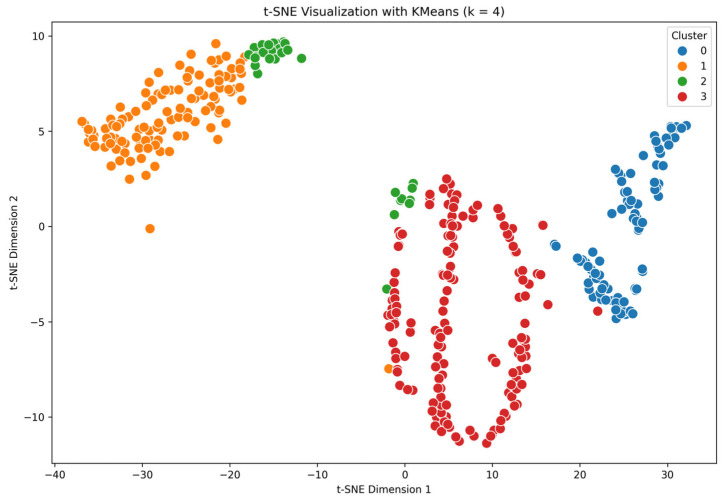
Clusters for k = 4.

**Figure A6:**
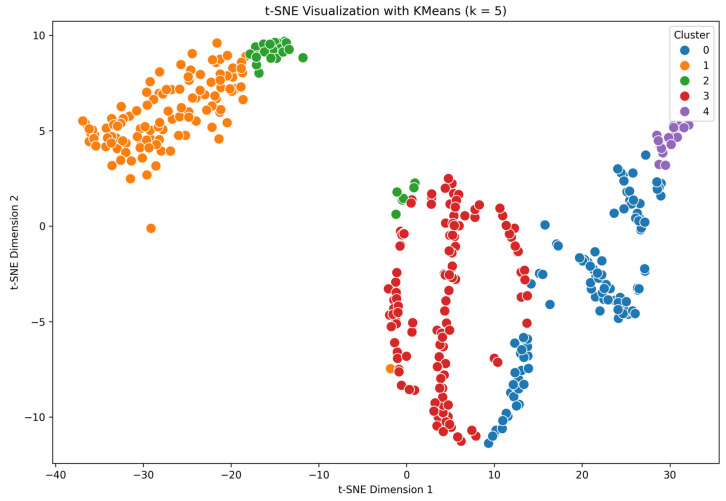
Clusters for k = 5.

While initial metrics suggested marginally stronger support for k = 4, visual inspection of t-SNE dimensionality reduction plots revealed a distinct patient subpopulation isolated in k = 5 solutions (Appendix B, Figure A6 upper right corner) that remained integrated within a larger cluster in k = 4 configurations (Appendix B, Figure A5). Clinical characterization of this subpopulation confirmed a distinctive phenotype (predominantly male, high smoking intensity, rural residence) with potential clinical relevance. Therefore, we selected the k = 5 solution for definitive analysis based on both statistical and clinical considerations.

We systematically grouped features by physiological systems according to ICD-10 anatomical classifications to enhance interpretability. Cluster characterization employed descriptive statistics (mean, standard deviation, and frequency) for both individual variables and feature ensembles to identify discriminative characteristics across the five identified patient subgroups.

### 2.4. Software Used

All statistical analyses were conducted using Python (version 3.11.12) within a validated computational environment. Data manipulation and preprocessing were executed using NumPy (2.0.2) and Pandas (2.2.2) libraries to ensure reproducible data transformation workflows. Statistical inference was performed via SciPy (1.15.3) and StatsModels (0.14.4), employing appropriate parametric and non-parametric methodologies based on data distribution characteristics.

The k-means clustering algorithm and associated validation metrics were implemented using the Scikit-learn package (version 1.6.1), with hyperparameter optimization conducted via cross-validation procedures. Matplotlib (3.10.0) and Seaborn (0.13.2) libraries were used to visualize multidimensional data and resultant clusters, employing standardized palettes for consistent interpretation across graphical outputs.

### 2.5. GenAI

The authors utilized Claude 3.7 Sonnet, a large language model developed by Anthropic, and ChatGPT-4-turbo, an AI-powered large language model developed by OpenAI, as writing assistance tools to enhance the manuscript’s structural organization and clarity of presentation. These tools were employed exclusively for linguistic refinement and formatting consistency, with all scientific content, interpretations, and conclusions determined solely by the authors.

All computational analyses were conducted in Google Colab, incorporating Google’s Gemini (2.2 Flash) AI-assisted code suggestion functionality. The programming investigator systematically evaluated these automated coding suggestions for validity and methodological soundness, with selective implementation at the discretion of the human. All implemented code underwent rigorous validation to ensure computational reproducibility and scientific integrity and can be found at https://colab.research.google.com/drive/1Xzg8qbUyBNZTzOwmIaT56Xq6JNrgT7P1?usp=sharing (accessed on 9 July 2025).

## 3. Results

### 3.1. General Patient Characteristics

The demographic profile of lung cancer patients admitted to our center underwent significant changes over the study period (Table 1), with a notable increase in annual admission rates in recent years, supporting the subjective observations investigated in Objective 1.

The median admissions per year increased from 385.5 cases (2013–2020) to 604.0 cases (2022–2024), representing a 57% increase that is less influenced by outlier years compared to the mean. This metric provides a robust measure of central tendency for our temporal analysis, particularly given the COVID-19 disruption in 2020–2021. The relationship between total admissions and unique patients reveals important changes in hospitalization patterns, with the rate of patients requiring multiple admissions increasing from 13.17% pre-pandemic to 20.35% post-pandemic. The annual rate of patients with multiple admissions more than doubled, from 41.38 patients per year to 100.33 patients per year, indicating increased disease complexity that requires iterative management approaches.

Patient age distributions shifted toward older populations, with median age increasing from 64.0 years (2013–2020) to 67.0 years (2022–2024). The age distribution became more negatively skewed in recent years (skewness: −0.37 to −0.70), indicating a more compressed distribution with fewer younger patients. This demographic shift aligns with regional population aging trends detailed in Appendix C.1, where substantial cohorts in the 65–79 age range create an expanding at-risk population for lung cancer development.

**Figure 1:**
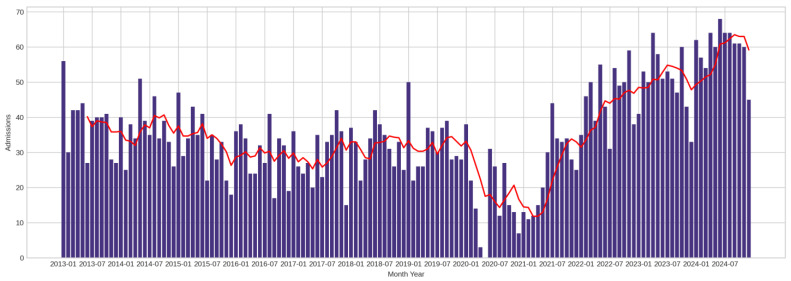
Admission rates 2013–2024 for lung cancer patients. The running average of six periods is presented in red.

From a visual standpoint, admission rates (Figure 1) demonstrate a clear temporal pattern consistent with national epidemiological trends. The data reveal a declining trajectory from 2013 to 2020, aligning with Romania’s reported downward trend from 41.2 per 100,000 inhabitants in 2010 to 38.8 per 100,000 in 2019. However, beginning in 2021, a sharp reversal occurs with consistent year-over-year increases through 2024. This pattern mirrors national data showing 7512 new cases in 2019 versus 11,857 cases in 2022, representing a 58% increase that contradicts the previously declining trend. While increased smoking rates in Romania (from 25.7% in 2014 to 27.3% in 2019, Appendix C.2) may contribute to this reversal, the rapid temporal onset suggests additional contributing factors beyond smoking exposure changes, which typically require 15–20 years to manifest as cancer incidence. The post-2021 increase is likely due to the convergence of delayed pandemic diagnoses, enhanced CT utilization, improved detection practices, and demographic aging effects in our regional population (See Appendix C.1 for details on aging effects).

**Figure 2:**
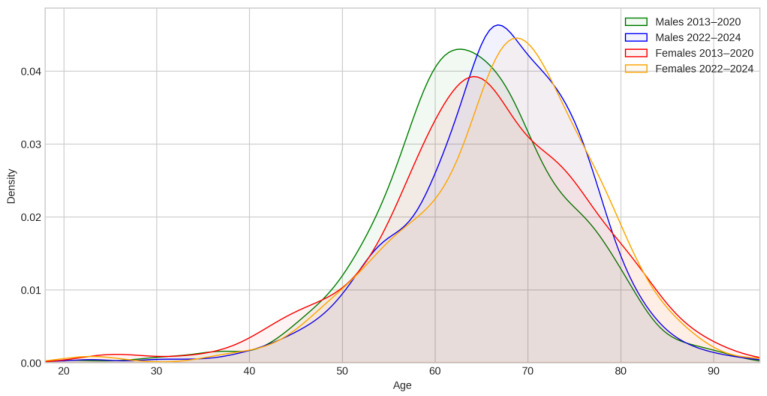
Age–sex distributions of lung cancer patients: Males–females distribution during two time intervals separated by the COVID-19 pandemic (2013–2020), (2022–2024).

In order to highlight that, in Figure 2, separate distributions were plotted for each distinct period.

### 3.2. Tumor Pathology and Immunohistochemistry Results

As stated, the tumor pathology and immunohistochemistry results are not performed locally and, therefore, are not available in the general patient database. Of the selected patients with those results, only 398 were collected over a 2-year period (2023–2024).

#### 3.2.1. General Characteristics

The smaller cohort parameters are presented in Table 2. For the active and former smokers, the median smoking intensity was 42.31 packages/year, and the maximum smoking intensity was 200 packages/year.

**Figure 3:**
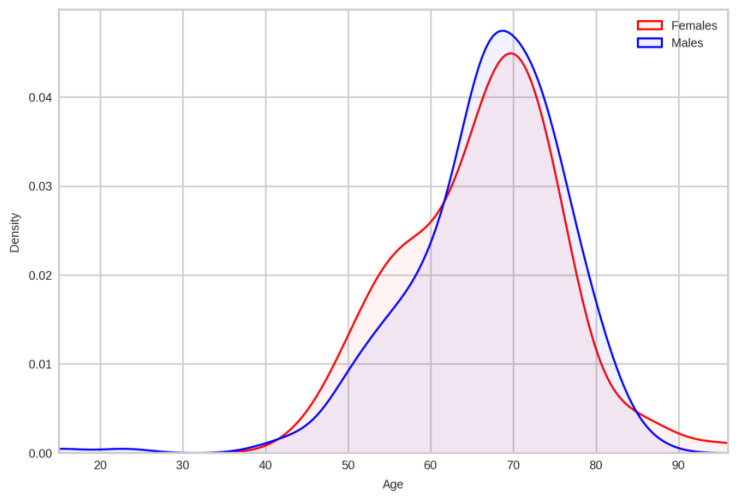
Pathology assessment cohort age distribution: females–males.

The age variation in this group (Figure 3) resembles the most recent age variation shown in Figure 2.

Comparison of age distributions between the smaller pathology-assessed cohort and general population data, stratified by sex, revealed no statistically significant differences. For males, both the Kolmogorov–Smirnov (K-S) test (D = 0.0603, *p* = 0.3521) and the Mann–Whitney U test (U = 175,912.50, *p* = 0.2621) indicated similar age distributions between the datasets. Similarly, for females, the K-S test (D = 0.0875, *p* = 0.4242) and the Mann–Whitney U test (U = 35,381.00, *p* = 0.2327) also showed similar age distributions. In all instances, *p*-values exceeded the 0.05 significance level, supporting the conclusion of similar age distributions across datasets for both sexes. Therefore, we can safely assume that our smaller cohort mirrors the whole 2022–2024 cohort.

#### 3.2.2. Histopathological Classification

Biopsy specimens were obtained from 373 patients (93.72% of the cohort). The remaining 25 patients (6.28%) had diagnoses established through alternative methods such as cytology or clinical–radiological correlation. The primary histological classification (Table 3) revealed adenocarcinoma as the most common subtype (33.7% of the total cohort), followed by squamous cell carcinoma (22.6%) and small cell lung cancer (8.3%). A significant proportion of cases (20.4%) remained unclassified due to diagnostic challenges or insufficient tissue sampling.

A detailed subtype analysis (Appendix B, Table A4) showed that among adenocarcinomas, acinar predominant (20.9% of ADK cases) and solid with mucin production (18.7% of ADK cases) were the most frequent patterns. However, 44.0% of adenocarcinomas could not be further subtyped due to limited tissue or mixed patterns. Similarly, among squamous cell carcinomas, 77.8% remained unspecified for keratinization status, reflecting the challenges of comprehensive subtyping in routine clinical practice.

#### 3.2.3. Immunohistochemistry and Molecular Characteristics

Immunohistochemistry testing was performed on 255 specimens (68.36% of biopsied cases). Biomarker testing rates and results (Table 4) varied considerably across different markers.

### 3.3. Cluster Analysis

The k-means clustering algorithm successfully identified five distinct patient clusters within our cohort of patients with lung cancer (n = 398).

To visualize the multidimensional clustering results and validate cluster separation, t-distributed Stochastic Neighbor Embedding (t-SNE) was employed for dimensionality reduction in the 761-feature dataset. The t-SNE algorithm, although computationally intensive, preserves local neighborhood relationships and projects high-dimensional patient data onto a two-dimensional plane for visualization.

This gradient visualization validates the biological relevance of our clustering approach, demonstrating that unsupervised machine learning successfully identified smoking-related disease stratification without explicit programming for these relationships.

**Figure 4:**
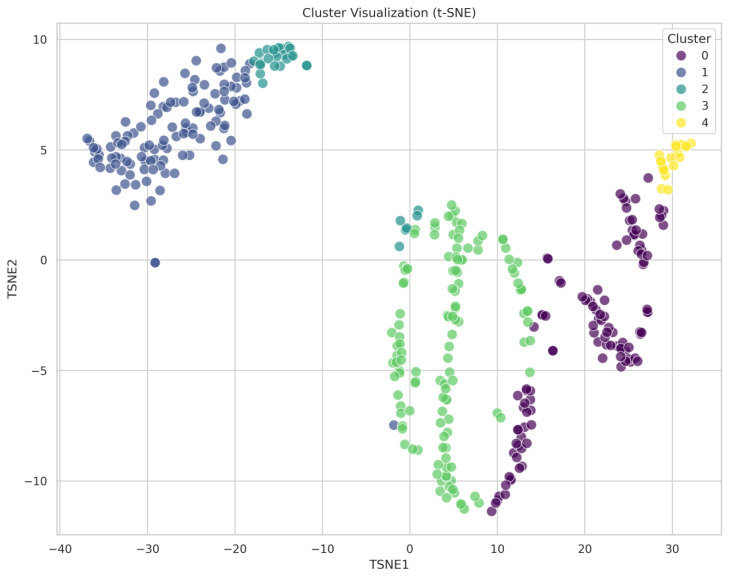
t-SNE visualization of five-cluster solution.

Figure 4 demonstrates the spatial distribution of the five identified clusters in reduced dimensional space. The t-SNE plot reveals distinct cluster boundaries with minimal overlap, confirming the validity of the k-means clustering approach. On the left are non-smokers, and as we go on the right, smoker clusters emerge. Cluster 4 (Extreme Heavy Smokers) appears as a well-separated outlier population in the upper portion of the plot, consistent with its extreme clinical phenotype. Also, in the smoking section, Clusters 0 and 3 (Heavy Smoking Males and Active Smokers) show spatial proximity, reflecting their shared smoking-related characteristics while maintaining distinct boundaries. Cluster 1 (Elderly Never-Smokers) occupies a discrete region, emphasizing its unique demographic and behavioral profile. Cluster 2 (Young Mixed-Gender Cohort) is the smallest group, split between smokers and non-smokers, with a higher proportion towards the latter.

Analysis of variance testing revealed highly significant differences among clusters across multiple clinical variables, with the top discriminative features demonstrating *p*-values < 0.001, confirming meaningful clinical differentiation between identified subgroups (see Table 5).

Smoking intensity (pack-years) represented the most discriminative feature (*p* < 0.001) in our analysis, demonstrating clear stratification across clusters.

**Figure 5:**
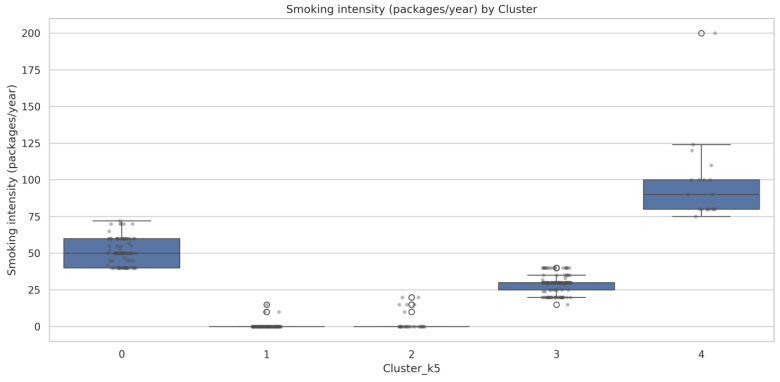
Smoking-intensity distribution by cluster.

Figure 5 illustrates the smoking-exposure gradient across clusters, revealing a clear biological gradient from never-smokers to extreme heavy smokers:

Cluster 1 shows minimal smoking exposure (mean 0.3 ± 1.9 pack-years), predominantly representing never-smokers with occasional light former smokers.

Cluster 2 demonstrates low–moderate exposure (mean 3.3 ± 6.7 pack-years), consistent with mixed never-smoker and light-smoker populations.

Cluster 3 exhibits a moderate smoking intensity, representing the typical active smoker population.

Cluster 0 shows high smoking exposure (mean 50.3 ± 8.7 pack-years), characteristic of heavy-smoking males.

Cluster 4 demonstrates extreme smoking intensity (mean 97.3 ± 28.8 pack-years), representing the most heavily exposed subpopulation in our cohort.

Since age emerged as one of the most statistically significant discriminative features (*p* < 0.001), it warrants a detailed examination of age distribution patterns.

**Figure 6:**
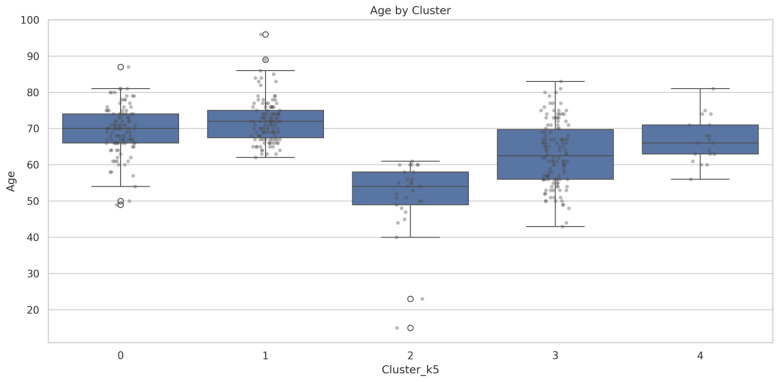
Age distribution by cluster. Pairwise comparisons using Mann–Whitney U tests showed significant age differences between most clusters: Cluster 0 vs. 1 (*p* < 0.01), Cluster 0 vs. 2 (*p* < 0.001), Cluster 0 vs. 3 (*p* < 0.001), Cluster 1 vs. 2 (*p* < 0.001), Cluster 1 vs. 3 (*p* < 0.001), Cluster 1 vs. 4 (*p* < 0.01), Cluster 2 vs. 3 (*p* < 0.001), and Cluster 2 vs. 4 (*p* < 0.001). Only Cluster 0 vs. 4 (*p* = 0.235) and Cluster 3 vs. 4 (*p* = 0.057) were not statistically significant.

Figure 6 presents the age distribution across the five clusters, revealing distinct age profiles that align with clinical expectations:

Cluster 2 demonstrates the youngest patient population (mean 51.0 ± 10.4 years, median age 54 years (IQR: 49–58)), with the widest age distribution, suggesting diverse etiological factors in younger lung cancer patients.

Cluster 1 exhibits the oldest patient cohort (mean 72.0 ± 5.9 years, median age 72 years (IQR: 67–75)), with the narrowest distribution, consistent with age-related lung cancer development in never-smokers.

Clusters 0 (median age 70 years (IQR: 66–74), 3 (median age 62.5 years (IQR: 56–70)), and 4 (median age 66 years (IQR: 63–71)) show intermediate age ranges, with overlapping but distinct distributions, reflecting smoking-related lung cancer patterns across different age groups.

The age stratification supports epidemiological evidence for distinct lung cancer pathogenesis pathways, with never-smoker disease occurring later in life, while smoking-related cancers demonstrate broader age ranges corresponding to variable smoking initiation and duration patterns.

The complete histological analysis (Appendix B, Table A3) reveals distinct cluster-specific patterns. Cluster 4 (Extreme Heavy Smokers) demonstrated the highest rates of squamous cell carcinoma (47.4%) and neuroendocrine differentiation (5.3%), consistent with heavy smoking-related histological changes. Conversely, Cluster 1 (Elderly Never-Smokers) showed the lowest NSCLC rate (52.2%) and paradoxically the highest SCLC rate (9.6%), suggesting alternative pathogenic mechanisms in this population. Large cell carcinoma was rare across all clusters (≤1.8%), while neuroendocrine differentiation was most prevalent in heavy smoking populations (Clusters 3–4: 4.0–5.3%).

Upon further examination of the clusters’ unique clinical, demographic, and pathological characteristics, several key characteristics emerged based on their highest mean values.

Cluster 0: Heavy Smoking Males (n = 109, 27.4%)

Clinical Profile: Predominantly male patients (88.1%) with the highest smoking intensity (50.3 ± 8.7 pack-years) and active smoking prevalence (69.7%). This cluster demonstrated the most aggressive smoking exposure pattern in our cohort.

Key Characteristics:

- Demographics: 89.0% male, mean age 69.6 ± 6.5 years, median age 70 years (IQR: 66–74);
- Smoking: 69.7% active smokers, mean 50.3 pack-years exposure;
- Pathology: 73.4% NSCLC, 41.3% adenocarcinoma;
- Molecular: 36.7% EGFR negative, 29.4% PD-L1 positive;
- Comorbidities: 33.9% acute infection with COPD (J44.0), 27.5% anemia (D63.0);
- Geography: 31.2% from outside Timis County.

Cluster 1: Elderly Never-Smokers (n = 115, 28.9%)

Clinical Profile: The second largest cluster, characterized by predominantly never-smoking elderly patients with the lowest smoking intensity and the highest proportion of female patients among the smoking-related clusters.

Key Characteristics:

- Demographics: 53.9% male, mean age 72.0 ± 5.9 years, median age 72 years (IQR: 67–75) (oldest cohort, significantly age different than all others);
- Smoking: 78.3% never smokers, mean 0.3 pack-years;
- Pathology: 52.2% NSCLC, 24.3% adenocarcinoma;
- Molecular: 60.0% PD-L1 testing absent, 20.9% EGFR negative;
- Disease stage: 44.3% metastatic disease (highest rate);
- Geography: Predominantly local Timis County residents.

Cluster 2: Young Mixed-Gender Cohort (n = 29, 7.3%)

Clinical Profile: The smallest cluster, distinguished by the youngest age and balanced gender distribution, with moderate never-smoking prevalence and a unique geographic distribution pattern.

Key Characteristics:

- Demographics: 51.7% male, mean age 51.0 ± 10.4 years, median age 54 years (IQR: 49–58) (youngest cohort, significantly different age from all other clusters);
- Smoking: 62.1% never smokers, mean 3.3 pack-years;
- Pathology: 48.3% NSCLC;
- Molecular: 82.8% EGFR testing absent, 79.3% ALK testing absent;
- Comorbidities: 44.8% hypertension (I10), 34.5% iron deficiency anemia (D53.9);
- Geography: 48.3% from outside Timis County, 41.4% from rural areas.

Cluster 3: Active Smokers with Comprehensive Testing (n = 126, 31.7%)

Clinical Profile: The largest cluster, characterized by high active smoking rates and the most comprehensive immunohistochemical testing coverage, represents a well-characterized patient population.

Key Characteristics:

- Demographics: Mean age 63.0 ± 8.7 years, median age 62.5 years (IQR: 56–70);
- Smoking: 61.9% active smokers, 38.1% former smokers, 0% never smokers;
- Pathology: 54.0% NSCLC;
- Molecular: 57.9% underwent IHC testing (highest rate), 46.8% PD-L1 testing absent;
- Disease stage: 24.6% metastatic disease;
- Geography: Mixed distribution across Timis County.

Cluster 4: Extreme Heavy Smokers (n = 19, 4.8%)

Clinical Profile: This cluster represents an extreme phenotype with the highest smoking intensity, universal biopsy procurement, and predominantly rural male composition.

Key Characteristics:

- Demographics: 94.7% male, predominantly rural (52.6%), median age 66 years (IQR: 63–71);
- Smoking: Mean 97.3 ± 28.8 pack-years (highest intensity), 63.2% active smokers;
- Pathology: 100% biopsy obtained, 73.7% NSCLC, 47.4% squamous cell carcinoma;
- Molecular: 89.5% IHC testing performed;
- Comorbidities: 47.4% acute infection with COPD (J44.0), 47.4% acute respiratory failure (J96.0);
- Geography: Strong rural concentration.

### 3.4. Overview of Respiratory Disease Burden

Given the study’s focus on potential contributing factors to regional lung cancer patterns, we conducted a detailed analysis of specific respiratory comorbidities across the identified clusters. Five key respiratory conditions were examined: COVID-19 history, Chronic Obstructive Pulmonary Disease (COPD), asthma, emphysema, and bronchiectasis, based on ICD-10 diagnostic codes documented in patient records.

The results, presented in Table 6, are differentiated for each cluster.

## 4. Discussion

### 4.1. Principal Findings

This study confirms the suspected increase in lung cancer presentations at our center, with median annual admissions rising from 345.63 (2013–2020) to 624.33 (2022–2024), representing an 80.5% increase in case volume. Through the unsupervised k-means clustering analysis of comprehensive clinical, pathological, and geographical data from 398 patients, we identified five distinct lung cancer patient phenotypes that demonstrate clear biological and clinical relevance. These clusters exhibited significant stratification across smoking intensity (*p* < 0.001), age distribution (*p* < 0.001), and gender composition (*p* < 0.001), suggesting the presence of distinct disease pathways within our regional population. Notably, while three clusters represent smoking populations, the findings extend beyond conventional smoking-based risk stratification. The algorithm identified clinically meaningful subdivisions among smokers that traditional approaches might miss. Over a third of our cohort were never-smokers, distributed across two distinct age-stratified clusters, reflecting broader epidemiological shifts in our region, where tertiary-educated individuals show smoking rates of 29.4% compared to 19.0% in the EU27 (Annex C).

The clustering analysis revealed a clear smoking exposure gradient from never-smokers (Cluster 1: 0.3 ± 1.9 pack-years) to extreme heavy smokers (Cluster 4: 97.3 ± 28.8 pack-years), demonstrating the algorithm’s ability to identify clinically meaningful patient stratifications without explicit programming for these relationships. Notably, the identification of Cluster 4 as a distinct outlier population of extreme heavy smokers with a unique geographic concentration (52.6% rural) and clinical characteristics (100% biopsy procurement, 94.7% male) suggests that region-specific factors warrant further investigation.

### 4.2. Temporal Trends and Contributing Factors

#### 4.2.1. Increased Case Volume: Multiple Contributing Factors

The number of monthly lung cancer cases increased significantly from 28.80 (2013–2020) to 52.03 (2022–2024), representing an 80.5% increase (t = −11.17, *p* < 0.001). This pattern aligns with national Romanian data, showing a decline in incidence from 2 to 10 in 2019, followed by a sharp 58% increase by 2022, confirming broader epidemiological trends that extend beyond institutional factors.

Evidence for a True Epidemiological Shift: Total hospital admissions (Appendix C.3) increased by only 51.1% (from 15,223 to 23,002 annually), whereas admissions for lung cancer rose by 80.5%. This 1.6-fold difference, despite the absence of changes in catchment area, referral policies, or competing healthcare facilities, suggests disease-specific factors rather than general changes in healthcare utilization. No systematic approach was in place during this period. A screening program was implemented during this period.

**Figure A8:**
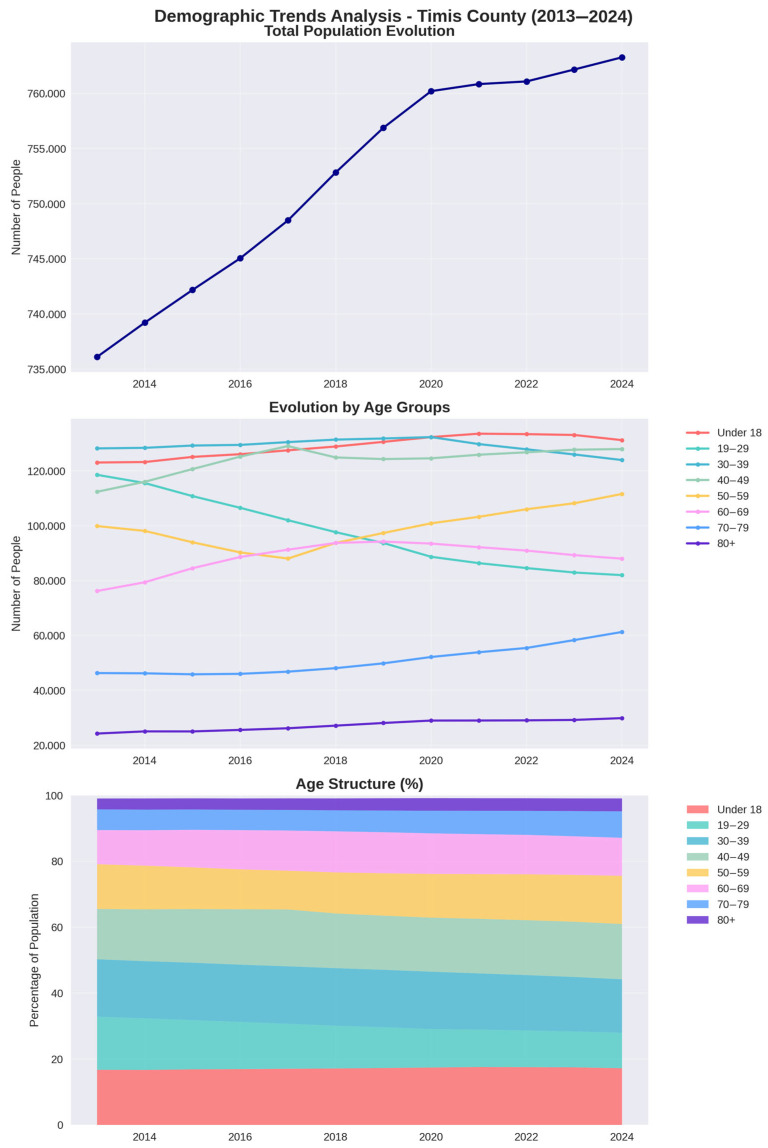
Demographic trends analysis—Timis County (2013–2024) (based on data from the National Statistics Institute).

**Figure A9:**
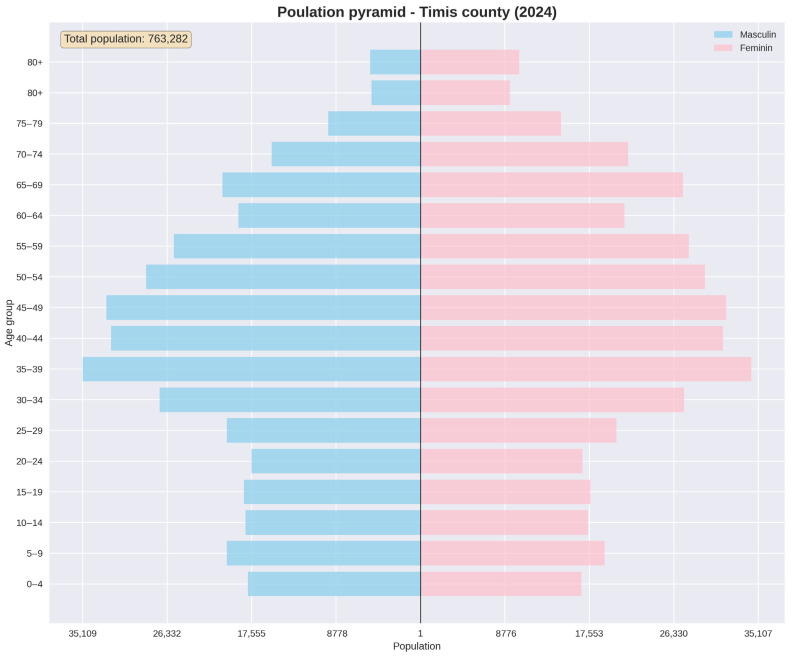
Population pyramid—Timis County (2024) (based on data from the National Statistics Institute).

**Figure A10:**
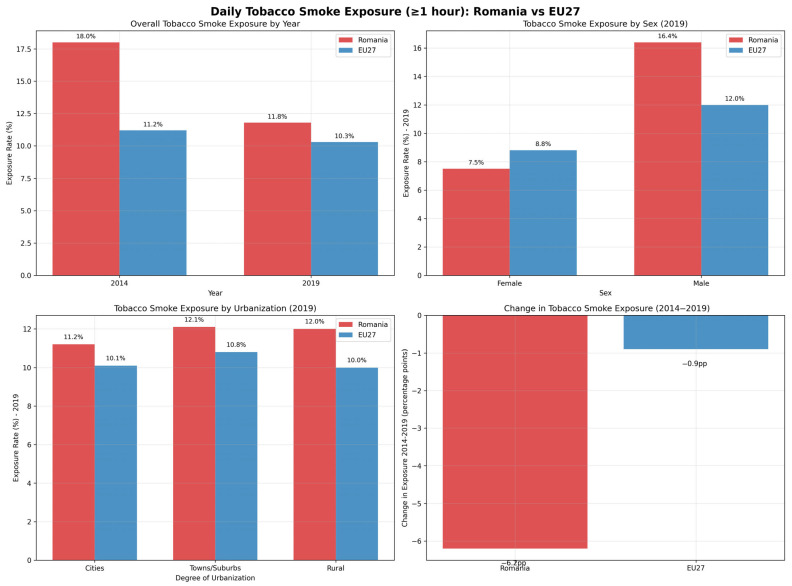
Daily tobacco smoke exposure (≥1 h): Romania vs. EU27. Data source: Eurostat Health Interview Survey (EHIS).

**Demographic Transitions**: The observed changes reflect convergent demographic shifts, with the median age increasing from 64.0 to 67.0 years, female representation rising from 25.35% to 30.43%, and the number of multi-admission patients doubling (from 41.38 to 100.33 patients per year). The 5-percentage-point increase in female lung cancer presentations aligns with global epidemiological trends showing convergent smoking patterns between genders over recent decades [20]. These shifts align with broader demographic transitions in Timiș County, as documented in Appendix C.1, where clear population-aging trends indicate substantial growth in the 60–79 age group—precisely the demographic most susceptible to lung cancer development (Appendix C, Figure A8 and Figure A9). The substantial population base in the 65–79 age range (approximately 15% of the total county population of 763,282) creates a large at-risk demographic that aligns with our Cluster 1 findings (Elderly Never-Smokers). Additionally, Romania’s smoking burden significantly exceeds European standards (Appendix C.2), with current smoking rates of 27.3% versus EU27’s 24.2%, and tertiary-educated individuals showing smoking rates of 29.4% compared to only 19.0% in EU27 (Appendix C, Figure A10). However, the rapid temporal onset suggests factors beyond smoking exposure changes alone, which typically require 15–20 years to manifest as cancer incidence.

**Healthcare System Factors**: Post-pandemic healthcare changes are likely to have contributed to delayed diagnoses, creating a “catch-up” effect, with patients presenting with more advanced diseases that require hospitalization [21,22]. This hypothesis is supported by higher metastatic disease rates (32.16%) in our smaller cohort. Recent international studies have shown that the COVID-19 pandemic led to a significant increase in chest CT utilization, particularly among hospitalized patients, for both the diagnosis and monitoring of COVID-19 and for other acute conditions [23,24,25]. This surge in imaging persisted into the post-pandemic era, resulting in a higher rate of incidental detection of pulmonary nodules and potential malignancies, particularly among older adults [24,26]. The increased use of chest CT scans during and after the pandemic likely contributed to the observed rise in lung cancer diagnoses at our institution, as more patients underwent imaging that could reveal previously undetected tumors. This phenomenon has been described as an “opportunistic screening effect” and is supported by multiple multicenter studies [23,24,26]. The systematic collection of pathology data starting in 2023 improved diagnostic completeness but coincided with broader changes in referral and diagnostic patterns.

This multifactorial pattern indicates genuine epidemiological shifts amplified by healthcare system adaptations rather than artifacts of detection or reporting changes.

#### 4.2.2. COVID-19 Impact: Limited Direct Association

Contrary to the hypothesized pandemic-related increases, a COVID-19 history was relatively uncommon across all clusters (3.45–8.26%), suggesting a limited direct causal relationship between SARS-CoV-2 infection and lung cancer development in our cohort, a relationship that remains incompletely understood or studied enough [27]. However, several caveats apply: the retrospective nature of COVID-19 diagnosis capture, potential underreporting of asymptomatic infections, and the relatively short latency period between the pandemic onset and our analysis window.

The low COVID-19 prevalence does not preclude indirect pandemic effects, such as delayed presentations, altered screening practices, or avoidance behaviors in healthcare that may have contributed to the observed case volume increase.

### 4.3. Clinical Significance of Identified Clusters

#### 4.3.1. Never-Smoker Phenotypes: Age-Related Disease Biology

The identification of Cluster 1 (Elderly Never-Smokers, comprising 28.9% of the cohort) as the largest never-smoker population with distinct characteristics (mean age, 72.0 years; 44.3% with metastatic disease) aligns with the emerging understanding of never-smoker lung cancer biology [28]. This population’s advanced age at presentation and high metastatic burden suggest different disease kinetics compared to smoking-related cancers, potentially reflecting slower-growing tumors with more extended subclinical periods or alternative biological drivers, such as environmental exposures or genetic predisposition [29].

Cluster 2 (Young Mixed-Gender Cohort, comprising 7.3% of the cohort) represents a particularly intriguing never-smoker subgroup characterized by the youngest mean age (51.0 years) and a balanced gender distribution. This cluster’s unique geographic distribution pattern (48.3% extra-jurisdictional) suggests potential referral patterns for rare or complex cases, possibly including hereditary cancer syndromes or occupational exposures requiring specialized evaluation. Furthermore, the spatial distribution can be interpreted as a manifestation of contemporary migratory phenomena characterized by the recent demographic repositioning of a youthful cohort into the county, primarily in response to more favorable economic conditions [30].

Although genetic predisposition has been linked to lung cancer among never-smoking elderly individuals, recent studies have also highlighted inherited susceptibility in young patients, especially in non-smokers. Familial aggregation and enriched prevalence of pathogenic germline variants with early-onset (non-smoking) lung cancer have been found in the research conducted by Carr and Cannon-Albright et al. [31,32,33]. In line with this view are our results in Cluster 2 (young, mixed-gender, largely non-smokers), indicating that genetic services and full molecular profiling may be indicated in this subset.

Recent comprehensive reviews and genomic studies have demonstrated a higher prevalence of germline alterations and distinct somatic mutation patterns in young lung cancer patients [34,35].

#### 4.3.2. Smoking-Related Disease Stratification

While three of the five clusters represent smoking populations potentially eligible for lung cancer screening, the granular stratification within smoking groups reveals clinically actionable heterogeneity beyond conventional risk models. The smoking-related clusters (0, 3, and 4) demonstrate clear dose–response relationships with distinct clinical phenotypes:

**Cluster 0 (Heavy Smoking Males)** represents the archetypal smoking-related lung cancer population with high active smoking rates (69.7%) and substantial pack-year exposure (50.3 ± 8.7). The predominant adenocarcinoma histology (41.3%) in this heavily smoking population may reflect modern smoking pattern changes and cigarette composition evolution [36].

**Cluster 3 (Active Smokers with Comprehensive Testing)** exhibited the highest immunohistochemical testing rates (57.9%), suggesting either more complex diagnostic requirements or enhanced access to molecular testing. This cluster’s universal smoking exposure (100% current/former smokers) with moderate pack-year burden represents patients likely to benefit from targeted therapy screening.

**Cluster 4 (Extreme Heavy Smokers)** emerges as a unique population that requires special attention. The extreme smoking intensity (mean 97.3 pack-years), rural concentration (52.6%), and universal biopsy procurement (100%) suggest a high-risk population with complex diagnostic requirements [37]. The high rates of acute respiratory complications (47.4% acute COPD exacerbation, 47.4% acute respiratory failure) indicate a substantial comorbidity burden requiring multidisciplinary management approaches [38].

#### 4.3.3. Geographic Clustering Patterns

The geographic distribution analysis reveals important regional disease patterns. The concentration of Cluster 4 (extreme heavy smokers) in rural areas (52.6%) suggests potential environmental or socioeconomic factors contributing to intensive smoking behaviors. Rural populations often experience delayed healthcare access, potentially explaining the extreme smoking exposures observed at the presentation [39].

Conversely, the substantial extra-jurisdictional representation (36.68% overall, 48.3% in Cluster 2) indicates our center’s role as a regional referral center, particularly for complex or rare cases. This referral pattern may influence the apparent epidemiology of disease and should be considered when interpreting regional cancer burden estimates.

Although three of the five identified clusters correspond to smoking populations typically eligible for lung cancer screening, our unsupervised clustering approach uncovered clinically and geographically meaningful heterogeneity within these groups. Specifically, the analysis revealed that extremely heavy smokers are concentrated in rural areas and bear a particularly high burden of respiratory comorbidities, while other smoking clusters differ by age, sex, and access to molecular testing. These geographic–clinical correlations reveal previously uncharacterized regional patterns that inform healthcare resource allocation beyond standard screening protocols, demonstrating that unsupervised clustering captures patient heterogeneity extending beyond conventional smoking-based risk stratification.

### 4.4. Respiratory Comorbidity Patterns

#### 4.4.1. COPD as a Unifying Factor

COPD prevalence across clusters (44.83–78.95%) substantially exceeds general population estimates, confirming the strong association between chronic airway disease and lung cancer development [40]. The highest COPD prevalence in Cluster 4 (78.95%) aligns with extreme smoking exposure and suggests shared pathophysiological pathways between chronic inflammation and carcinogenesis [41].

The relatively uniform COPD distribution across smoking-related clusters (66–79%) contrasts with the lower prevalence in the young mixed-gender cohort (44.83%), supporting distinct disease mechanisms in younger patients that may be less dependent on chronic inflammatory processes [42].

#### 4.4.2. Asthma Associations: Unexpected Patterns

Asthma prevalence showed an inverse correlation with smoking intensity, with the highest rates in never-smoker populations (Cluster 1: 15.65%) and the lowest in extreme smokers (Cluster 4: 5.26%) [43]. This pattern may reflect diagnostic challenges in distinguishing asthma from COPD in heavy smokers, smoking cessation recommendations for asthmatic patients, or potential protective effects of asthma medications against lung cancer development—a hypothesis requiring prospective investigation [44,45].

### 4.5. Molecular and Pathological Implications

The variable immunohistochemical and molecular testing rates across clusters (ranging from minimal in Cluster 2 to 89.5% in Cluster 4) highlight important healthcare delivery disparities. The youngest patients (Cluster 2) had the lowest testing rates despite potentially benefiting most from targeted therapies, while extreme heavy smokers (Cluster 4) received comprehensive testing despite historically lower targetable mutation rates [46].

EGFR positivity emerged as a highly significant discriminating factor (*p* < 0.001), indicating that molecular characteristics align with clinical clustering patterns, potentially reflecting distinct therapeutic response profiles. The observed EGFR mutation rate (7.87% of tested patients) appears consistent with European populations but warrants expansion to include larger, never-smoker cohorts where mutation rates typically exceed 15% [47,48]. Similarly, the 49.74% PD-L1 positivity rate across the tested patients suggests that substantial populations are potentially eligible for immunotherapy [49].

### 4.6. Study Strengths and Limitations

#### 4.6.1. Methodological Strengths

This study employed robust unsupervised machine learning techniques, along with comprehensive validation metrics, to identify naturally occurring patient clusters without making prior assumptions about disease classification. The integration of clinical, pathological, and geographical data enables multidimensional patient characterization, a feature rarely achieved in single-center studies.

The temporal analysis spanning 12 years provides sufficient power to detect epidemiological trends, while the detailed pathological characterization of recent patients enables molecular-level cluster validation.

#### 4.6.2. Acknowledged Limitations

**Retrospective Design**: The retrospective nature of this design limits causal inference and introduces potential selection biases, particularly regarding the availability of pathological data.

**Single-Center Analysis**: Although providing detailed regional characterization, single-center data may not be generalizable to broader populations or different healthcare systems.

**Missing Data**: Substantial missing anthropometric and radiological data required exclusion from the analysis, potentially affecting the cluster composition.

**Temporal Data Heterogeneity**: The asymmetric availability of pathological data (comprehensive only from 2023 onward) creates potential temporal confounding in cluster characteristics.

**Geographic Exposure Assessment**: Using residential addresses as proxies for environmental exposure oversimplifies complex mobility patterns and exposure histories.

**Environmental Exposure Assessment**: The limited availability of comprehensive environmental monitoring data, particularly for radon exposure and air quality metrics at patient-specific locations, prevented the integration of these established lung cancer risk factors into our clustering analysis. While regional data suggest elevated radon concentrations and our center has documented air pollution impacts on respiratory diseases, the spatial resolution and temporal coverage of available environmental data were insufficient for patient-level exposure modeling.

### 4.7. Clinical and Public Health Implications

#### 4.7.1. Personalized Medicine Applications

The identified clusters provide frameworks for tailored screening, diagnostic, and treatment approaches:

- **Never-smoker populations** (Clusters 1 and 2) may benefit from enhanced genetic counseling and familial risk assessment protocols, with age-stratified approaches reflecting distinct pathophysiological pathways.
- **Heavy smoking populations** (Clusters 0 and 4) require integrated pulmonary rehabilitation and smoking-cessation programs alongside cancer care.
- **Young patients** (Cluster 2) warrant comprehensive molecular profiling despite limited current testing rates.

#### 4.7.2. Healthcare Resource Allocation

The geographic concentration of high-risk populations (particularly rural extreme smokers) suggests targeted screening and prevention program deployment opportunities. Enhanced rural healthcare access and smoking-cessation resources may yield disproportionate cancer prevention benefits.

#### 4.7.3. Future Research Priorities

Future investigations should prioritize the following:

- Prospective validation of identified clusters in independent populations;
- Environmental exposure assessment beyond residential geocoding;
- Longitudinal outcomes analysis across cluster-specific treatment approaches;
- Expanded molecular profiling in never-smoker populations.

## 5. Conclusions

This analysis confirms significant temporal increases in lung cancer presentations at our regional center while identifying five distinct patient phenotypes with clear biological and clinical relevance. The successful application of unsupervised clustering techniques demonstrates the potential for data-driven patient stratification to inform personalized medicine approaches, revealing clear gradients in smoking exposure, age, comorbidity burden, and molecular characteristics.

The identification of extreme heavy smokers as a distinct rural population with unique healthcare needs, alongside never-smoker clusters with age-stratified disease patterns, provides actionable insights for targeted prevention and treatment strategies. While the history of COVID-19 has shown a limited direct association with the development of lung cancer, the complex interplay of pandemic-related healthcare disruptions likely contributed to the observed increases in case volume. Therefore, the observed increase is not due to a new screening program or a change in the consent policy, but rather to broader changes in the healthcare system and diagnostic practices following the pandemic.

These findings establish a foundation for prospective validation studies and suggest region-specific factors warranting further investigation in lung cancer epidemiology and personalized treatment approaches.

While three clusters represent smoking populations, the identification of clinically meaningful subdivisions within smoking groups and substantial never-smoker representation (37.24%) demonstrates that unsupervised clustering reveals patient heterogeneity extending beyond conventional smoking-based risk models.

The authors used ChatGPT-4-turbo, an AI-powered large language model developed by OpenAI, to improve the manuscript’s language and readability exclusively. All the scientific content, interpretations, and conclusions are based on the original work of the authors.

## Figures and Tables

**Table 1:** Demographic data for lung cancer patients, whole range (2013–2024), until COVID-19 (2013–2020), and post-COVID-19 (2022–2024). All float values have been rounded up to 2 decimals.

Category	Metric	2013–2024	2013–2020	2022–2024
Admissions	Total numbers	5145	2973	1873
	Mean admissions/year	428.75	371.62	624.33
	Median admissions/year	387.50	385.50	604.00
	Mean admissions/month	35.98	31.29	52.03
	Median admissions/month	35.00	33.00	53.00
Patients	Unique patients	4204	2514	1479
	Patients with multiple admissions	695	331	301
	Multiple admission rate	16.53%	13.17%	20.35%
	Repeat patients/year	57.92	41.38	100.33
Gender	Males	3740	2214	1303
	% males	72.69%	74.47%	69.57%
	Females	1405	759	570
	% females	27.31%	25.53%	30.43%
Age	Mean value	65.29	64.43	66.57
	Median value	66.00	64.00	67.00
	Age std	10.20	10.35	9.92
	Age min	18	19	18
	Age 25%	59	58	61
	Age 50%	66	64	67
	Age 75%	72	71	73
	Age max	95	94	95
	Age skewness	−0.50	−0.37	−0.70
	Age kurtosis:	1.12	1	1.59

**Table 2:** Demographic and geographic distribution of the pathology assessment cohort.

	Characteristic	Number (Total = 398)	Percentage
Gender	Male	282	70.85%
	Female	116	29.15%
Geographic Distribution *	Timisoara metropolitan core	99	24.87%
	Timis county—suburban	28	7.04%
	Timis county—rural areas	138	34.67%
	Outside Timis county	146	36.68%
Smoking Status	Never smoker	108	27.14%
	Active smoker	185	46.48%
	Former smoker	105	26.38%
Occupational Exposure	Respiratory Irritants Exposure	62	15.58%
Notable Comorbidities	Metastasis	128	32.16%
	COPD	263	66.08%
	Asthma	46	11.56%
	COVID (infection and history)	25	6.28%

* The sum of this category is larger than the total since some patients changed home addresses and were counted in multiple categories.

**Table 3:** Histopathological classification of lung cancers in the study cohort.

Histological Type	Count	% of Total Cohort (n = 398)	% of Biopsied Cases (n = 373)
Adenocarcinoma	134	33.7%	35.9%
Squamous Cell Carcinoma	90	22.6%	24.1%
Small Cell Lung Cancer	33	8.3%	8.8%
Large Cell Carcinoma	2	0.5%	0.5%
Not Otherwise Specified	33	8.3%	8.8%
Other/Unclassified	81	20.4%	21.7%

Biopsy specimens were obtained from 373 patients (93.7% of the cohort). The remaining 25 patients had diagnoses established through alternative methods.

**Table 4:** Immunohistochemistry and molecular methods for biomarker results. ALK (anaplastic lymphoma kinase), PD-L1 (programmed death-ligand 1), and EGFR (epidermal growth factor receptor).

Biomarker	Tested (n)	Positive	Negative	Testing Rate (% Biopsies)	Positivity Rate (% Tested)
ALK	134	2	132	35.92%	1.49%
PD-L1	189	94	95	50.67%	49.74%
EGFR	127	10	117	34.05%	7.87%

**Table 5:** Most statistically significant discriminative features.

Clinical Feature	*p*-Value	Clinical Domain
Smoking intensity (pack-years)	<0.001	Behavioral risk factor
Smoking status (Never/Active/Former) *	<0.001	Behavioral risk factor
Age	<0.001	Demographics
Gender (Male)	<0.001	Demographics
Breast Cancer History (C50.0)	0.0005	Comorbidity
Essential Thrombocythemia (D47.7)	0.0005	Hematological disorder
Coagulation Disorder (D68.9)	0.0005	Hematological disorder
Anxiety Disorder (F41.0)	0.0005	Psychiatric comorbidity
Sleep Apnea (G47.30)	0.0005	Respiratory comorbidity

* All three categories.

**Table 6:** Respiratory comorbidities across lung cancer patient clusters.

Condition	Cluster 0 (n = 109)	Cluster 1 (n = 115)	Cluster 2 (n = 29)	Cluster 3 (n = 126)	Cluster 4 (n = 19)
COPD (J44.0–J44.9)	68.81%	66.09%	44.83%	66.67%	78.95%
Asthma (J45.0–J45.9)	12.84%	15.65%	13.79%	7.14%	5.26%
Emphysema (J43.0–J43.9)	21.10%	9.57%	3.45%	17.46%	42.11%
Bronchiectasis (J47)	24.77%	18.26%	20.69%	21.43%	31.58%
COVID-19 (U07.1, U09.9)	8.26%	6.96%	3.45%	4.76%	5.26%

**Figure A1:**
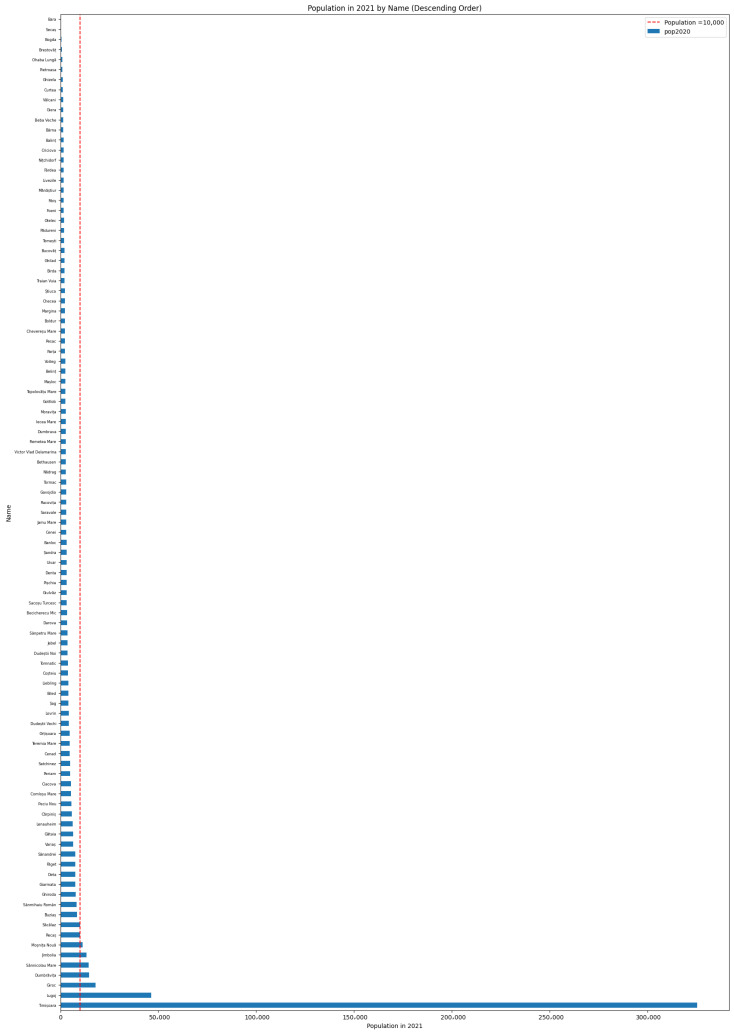
Population numbers for all the administrative units in Timis County. Cut-off rate at 10,000 people. Data corresponding to the 2021 national census.

**Figure A7:**
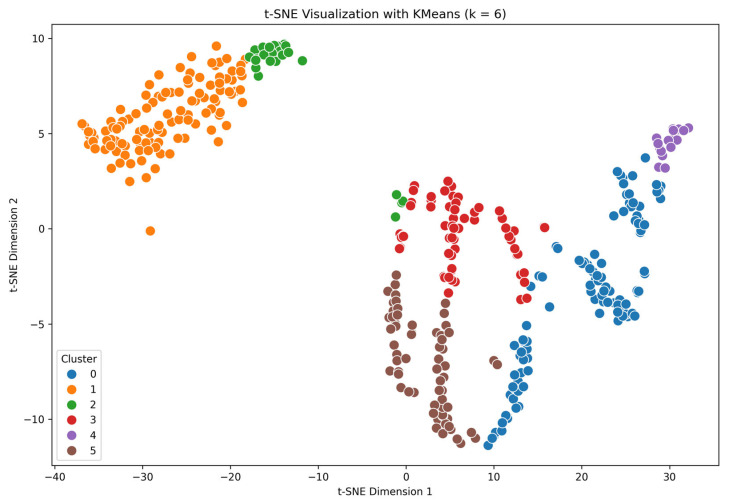
Clusters for k = 5.

**Figure A11:**
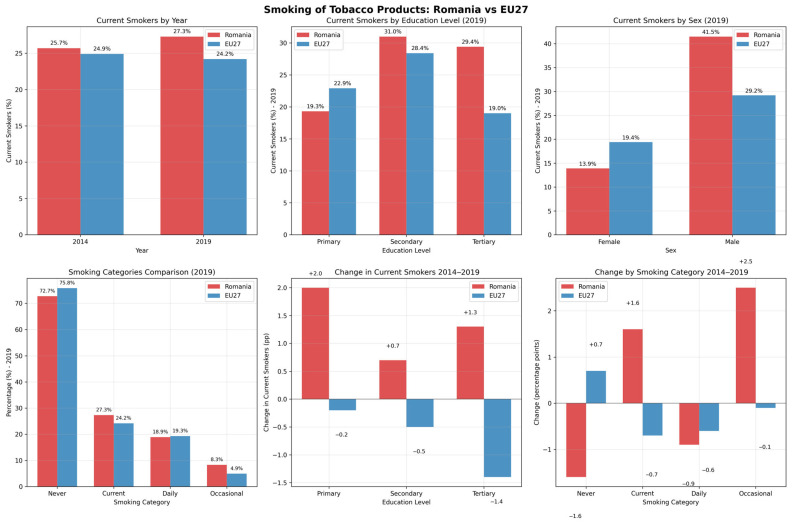
Smoking of tobacco products: Romania vs. EU27. Total Population = Never Smokers (NSM) + Current Smokers (SM_CUR). Current Smokers (SM_CUR) = Daily Smokers (SM_DAY) + Occasional Smokers (SM_OCC). Data source: Eurostat Health Interview Survey (EHIS).

**Figure A12:**
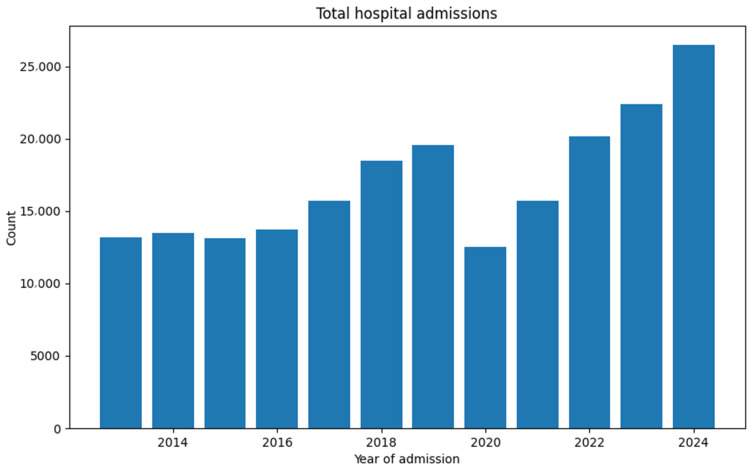
Total hospital admissions—Victor Babes University Hospital (2013–2024).

## Data Availability

The datasets generated and/or analyzed during the current study are not publicly available due to privacy restrictions (GDPR). However, anonymized datasets may be available upon reasonable request and with appropriate ethical approval. Requests to access the datasets should be directed to Dr. Oancea (oancea@umft.ro).

## Abbreviations

The following abbreviations are used in this manuscript:

ADK	Adenocarcinoma
AI	Artificial Intelligence
ALK	Anaplastic Lymphoma Kinase
COPD	Chronic Obstructive Pulmonary Disease
COVID-19	Coronavirus Disease 2019
CT	Computed Tomography
EGFR	Epidermal Growth Factor Receptor
GenAI	Generative Artificial Intelligence
ICD-10	International Classification of Diseases, 10th Revision
IHC	Immunohistochemistry
K-S	Kolmogorov–Smirnov
LCC	Large Cell Carcinoma
NOS	Not Otherwise Specified
NSCLC	Non-Small Cell Lung Cancer
PD-L1	Programmed Death-Ligand 1
SARS-CoV-2	Severe Acute Respiratory Syndrome Coronavirus 2
SCC	Squamous Cell Carcinoma
SCLC	Small Cell Lung Cancer
SNOMED CT	Systematized Nomenclature of Medicine—Clinical Terms
t-SNE	t-distributed Stochastic Neighbor Embedding
WHO	World Health Organization

## Appendix A

Our analysis identified four distinct classes of administrative-territorial units (UATs) in Timis County, each with specific demographic and spatial characteristics that influence lung cancer distribution patterns. This classification facilitates a more nuanced understanding of the data and informs targeted public health interventions.

Class 0: Metropolitan Core (Timisoara Municipality)

Timisoara exhibited a dominant pattern in the lung cancer case distribution, consistent with its demographic weight (comprising 33% of the county’s population while occupying only 1.5% of the county’s area) [8,9]. The metropolitan core demonstrated the highest absolute case numbers and population-adjusted rates, functioning as the epidemiological epicenter within the county.

Class 1: Sub-Urban Areas

This profile included UATs with approximately 10,000 residents or more, representing secondary urban centers or areas with significant demographic density. Based on the latest census data, this category comprised, in descending order of population: Lugoj, Giroc, Dumbrăvița, Sânnicolau Mare, Jimbolia, and Moșnița Nouă. Recaș and Săcălaz were also included in this category, given their proximity to the 10,000-resident threshold (200 and 500 persons below, respectively). In total, this profile encompassed eight UATs and displayed intermediate lung cancer rates with notable clustering patterns.

Class 2: Rural Areas

This class referred to UATs with populations below 10,000. This class encompassed the remaining 83 UATs, characterized by a more dispersed population.

Class 3: Outside Timis County

This class included the population that had home addresses outside Timis county. These might represent referrals or migratory patterns, but due to the retrospective nature of the study, it is hard to properly characterize them.

**Table A1:** Geographical patterns across lung cancer patient clusters.

Area	Cluster 0 (n = 109)	Cluster 1 (n = 115)	Cluster 2 (n = 29)	Cluster 3 (n = 126)	Cluster 4 (n = 19)
Timisoara main	28.44%	24.35%	13.79%	26.98%	10.53%
Timisoara sub-urban	9.17%	4.35%	0.00%	9.52%	5.26%
Rural Timis county	34.86%	33.91%	41.38%	30.95%	52.63%
Outside Timis county	31.19%	40.87%	48.28%	36.51%	31.58%

## Appendix B

**Table A2:** Quality scores.

K	Silhouette Score	Calinski–Harabasz Index	Davies–Bouldin Score
4	0.418590	370.444377	0.863661
5	0.413860	449.803775	0.838294
6	0.406199	426.908887	0.849540

**Table A3:** Complete pathological classification by cluster for k = 5.

Pathology	Cluster 0 (n = 109)	Cluster 1 (n = 115)	Cluster 2 (n = 29)	Cluster 3 (n = 126)	Cluster 4 (n = 19)
NSCLC	73.4%	52.2%	48.3%	54.0%	73.7%
SCLC	8.3%	9.6%	6.9%	7.9%	5.3%
Adenocarcinoma	41.3%	24.3%	31.0%	36.5%	31.6%
Squamous Cell	23.9%	22.6%	17.2%	19.0%	47.4%
NOS	11.0%	7.8%	3.4%	7.9%	5.3%
Large Cell Carcinoma	1.8%	0.0%	0.0%	0.0%	0.0%
Neuroendocrine	1.8%	0.9%	0.0%	4.0%	5.3%
No Biopsy	5.5%	7.0%	3.4%	7.9%	0.0%
Metastatic Disease	30.3%	44.3%	27.6%	24.6%	26.3%

**Table A4:** Detailed histopathological classification with subtypes.

Classification Level	Histological Type	Count	% of Total (n = 398)	% of Category
Major Types				
	Non-Small Cell Lung Cancer	236	59.3%	-
	Small Cell Lung Cancer	33	8.3%	-
	Not Otherwise Specified	33	8.3%	-
	No Tissue Diagnosis	96	24.1%	-
NSCLC Subtypes				
	Adenocarcinoma	134	33.7%	56.8%
	Squamous Cell Carcinoma	90	22.6%	38.1%
	Large Cell Carcinoma	2	0.5%	0.8%
	NSCLC Unspecified	10	2.5%	4.2%
Adenocarcinoma Subtypes				
	Acinar Predominant	28	7.0%	20.9%
	Solid with Mucin Production	25	6.3%	18.7%
	Lepidic Predominant	8	2.0%	6.0%
	Papillary Predominant	8	2.0%	6.0%
	Micropapillary Predominant	6	1.5%	4.5%
	ADK Subtype Unspecified	59	14.8%	44.0%
Squamous Cell Subtypes				
	With Keratinization	7	1.8%	7.8%
	Non-Keratinizing	13	3.3%	14.4%
	SCC Subtype Unspecified	70	17.6%	77.8%
Other Features				
	Neuroendocrine Differentiation	9	2.3%	-
	Glandular Differentiation	10	2.5%	-
	Metastatic Disease Confirmed	128	32.2%	-

## Appendix C

### Appendix C.2. Smoking Patterns

Romania Daily Exposure ≥ 1 h Statistics

2014 Statistics:

     Records available: 252;

     Mean exposure: 17.82%;

     Median exposure: 17.90%;

     Min exposure: 1.50%;

     Max exposure: 35.60%;

     Standard deviation: 7.65%.

2019 Statistics:

     Records available: 204;

     Mean exposure: 11.33%;

     Median exposure: 11.20%;

     Min exposure: 0.50%;

     Max exposure: 26.00%;

     Standard deviation: 5.36%.

Breakdown by Sex:

     Female: 2014 avg = 12.21%, 2019 avg = 7.59%;

     Male: 2014 avg = 23.45%, 2019 avg = 15.10%;

     Total: 2014 avg = 17.81%, 2019 avg = 11.30%.

Breakdown by Degree of Urbanization:

     Cities (densely populated): 2014 avg = 17.02%, 2019 avg = 11.04%;

     Towns/suburbs (intermediate): 2014 avg = 17.81%, 2019 avg = 10.99%;

     Rural areas (thinly populated): 2014 avg = 18.58%, 2019 avg = 11.90%;

     Total: 2014 avg = 17.88%, 2019 avg = 11.39%.

Romania Smoking Statistics

2014 Statistics (All Education Levels):

     Never Smoked: 74.3%;

     Current Smokers: 25.7%;

     Daily Smokers: 19.8%;

     Occasional Smokers: 5.8%.

2019 Statistics (All Education Levels):

     Never Smoked: 72.7%;

     Current Smokers: 27.3%;

     Daily Smokers: 18.9%;

     Occasional Smokers: 8.3%.

Current Smokers by Education Level:

2014:

     Primary/Lower Secondary (ISCED 0–2): 17.3%;

     Upper Secondary/Post-Secondary (ISCED 3–4): 30.3%;

     Tertiary Education (ISCED 5–8): 28.1%.

2019:

     Primary/Lower Secondary (ISCED 0–2): 19.3%;

     Upper Secondary/Post-Secondary (ISCED 3–4): 31.0%;

     Tertiary Education (ISCED 5–8): 29.4%.

Current Smokers by Sex (All Education, 2019):

     Female: 13.9%;

     Male: 41.5%.

EU27 Daily Exposure ≥1 h Statistics

2014 Statistics:

     Records available: 204;

     Mean exposure: 11.57%;

     Median exposure: 12.45%;

     Min exposure: 2.70%;

     Max exposure: 17.60%;

     Standard deviation: 3.41%.

2019 Statistics:

     Records available: 204;

     Mean exposure: 10.72%;

     Median exposure: 10.90%;

     Min exposure: 2.70%;

     Max exposure: 16.60%;

     Standard deviation: 2.67%.

Breakdown by Sex:

     Female: 2014 avg = 9.94%, 2019 avg = 9.33%;

     Male: 2014 avg = 13.22%, 2019 avg = 12.12%;

     Total: 2014 avg = 11.54%, 2019 avg = 10.70%.

Breakdown by Degree of Urbanization:

Cities (densely populated): 2014 avg = 11.93%, 2019 avg = 10.21%;

     Towns/suburbs (intermediate): 2014 avg = 10.92%, 2019 avg = 11.10%;

     Rural areas (thinly populated): 2014 avg = 11.86%, 2019 avg = 10.89%;

     Total: 2014 avg = 11.57%, 2019 avg = 10.67%.

     EU27 Smoking Statistics

2014 Statistics (All Education Levels):

     Never Smoked: 75.2%;

     Current Smokers: 24.8%;

     Daily Smokers: 19.8%;

     Occasional Smokers: 5.0%.

2019 Statistics (All Education Levels):

     Never Smoked: 75.8%;

     Current Smokers: 24.2%;

     Daily Smokers: 19.3%;

     Occasional Smokers: 4.9%.

Current Smokers by Education Level:

2014:

     Primary/Lower Secondary (ISCED 0–2): 22.9%;

     Upper Secondary/Post-Secondary (ISCED 3–4): 28.8%;

     Tertiary Education (ISCED 5–8): 20.4%.

2019:

     Primary/Lower Secondary (ISCED 0–2): 22.9%;

     Upper Secondary/Post-Secondary (ISCED 3–4): 28.4%;

     Tertiary Education (ISCED 5–8): 19.0%.

Current Smokers by Sex (All Education, 2019):

     Female: 19.4%;

     Male: 29.2%.

### Appendix C.3. Institutional Context—Total Hospital Admissions

**Table A5:** Total hospital admissions—Victor Babes University Hospital (2013–2024).

Year	Total Admissions	Year-Over-Year Change
2013	13,196	
2014	13,468	2.06%
2015	13,146	−2.39%
2016	13,748	4.58%
2017	15,679	14.05%
2018	18,497	17.97%
2019	19,530	5.58%
2020	12,523	−35.88%
2021	15,682	25.23%
2022	20,149	28.48%
2023	22,381	11.08%
2024	26,477	18.30%

Pre-pandemic average (2013–2020): 15,223 admissions/year;

Post-pandemic average (2022–2024): 23,002 admissions/year;

Overall increase: 51.1%.

## References

[1] Zhou J., Xu Y., Liu J., Feng L., Yu J., Chen D. (2024). Global burden of lung cancer in 2022 and projections to 2050: Incidence and mortality estimates from GLOBOCAN. Cancer Epidemiol..

[2] Bray F., Laversanne M., Sung H., Ferlay J., Siegel R.L., Soerjomataram I., Jemal A. (2024). Global cancer statistics 2022: GLOBOCAN estimates of incidence and mortality worldwide for 36 cancers in 185 countries. CA Cancer J. Clin..

[3] ANALIZA-SITUATIE-CANCER-2022.pdf—Institutul Național de Sănătate Publică. 21 June 2022. https://insp.gov.ro/download/analiza-situatie-cancer-2022-pdf/.

[4] https://insp.gov.ro/download/cnsisp/Fisiere-de-pe-site-CNSISP/mortalitatea_generala/Mortalitatea-generala-2020.pdf.

[5] Raport_ss_23_final.pdf. https://www.dsptimis.ro/promovare/raport_ss_23_final.pdf.

[6] Lynch C.M., van Berkel V.H., Frieboes H.B. (2017). Application of unsupervised analysis techniques to lung cancer patient data. PLoS ONE.

[7] Andrijanova A., Bugovecka L., Isajevs S., Erts D., Malinovskis U., Liepins A. (2025). Machine Learning for Lung Cancer Subtype Classification: Combining Clinical, Histopathological, and Biophysical Features. Diagnostics.

[8] International Agency for Research on Cancer (IARC) (2021). Thoracic Tumours (WHO Classification of Tumours, 5th Edition, Volume 5).

[9] Recording Smoking Status|Ministry of Health, NZ 23 January 2025. https://www.health.govt.nz/strategies-initiatives/programmes-and-initiatives/smokefree-2025/information-for-practitioners-of-patients-who-are-quitting-smoking/recording-smoking-status.

[10] 2024 GINA Main Report. Global Initiative for Asthma—GINA. https://ginasthma.org/2024-report/.

[11] 2025 GOLD Report. Global Initiative for Chronic Obstructive Lung Disease—GOLD. https://goldcopd.org/2025-gold-report/.

[12] World Health Organization (WHO). https://www.who.int.

[13] Home Page|United States Preventive Services Taskforce. https://www.uspreventiveservicestaskforce.org/uspstf/.

[14] Current Biomarkers in Non-Small Cell Lung Cancer—The Molecular Pathologist’s Perspective. https://www.mdpi.com/2075-4418/15/5/631.

[15] Județul Timiș. https://ro.wikipedia.org/wiki/Jude%C8%9Bul_Timi%C8%99.

[16] (2025). Timișoara. https://ro.wikipedia.org/w/index.php?title=Timi%C8%99oara&oldid=16907448.

[17] Trusculescu A.A., Ancusa V.M., Pescaru C.C., Wellmann N., Fira-Mladinescu C., Oancea C.I., Fira-Mladinescu O. (2024). A Multifaceted Exploration of Status Asthmaticus: A Retrospective Analysis in a Romanian Hospital. J. Clin. Med..

[18] Trusculescu A.A., Ancusa V.M., Burducescu A., Pescaru C.C., Trăilă D., Wellmann N., Fira-Mladinescu O., Oancea C.I. (2025). Age-Related Variations and Seasonal Influences: A Network Analysis of Comorbidities in Asthma Hospitalizations (2013–2023). J. Clin. Med..

[19] Tollefsen T., Cinelli G., Bossew P., Gruber V., De Cort M. (2014). From the European indoor radon map towards an atlas of natural radiation. Radiat. Prot. Dosim..

[20] Siegel R.L., Kratzer T.B., Giaquinto A.N., Sung H., Jemal A. (2025). Cancer statistics, 2025. CA A Cancer J. Clin..

[21] Moynihan R., Sanders S., Michaleff Z.A., Scott A.M., Clark J., To E.J., Jones M., Kitchener E., Fox M., Johansson M. (2021). Impact of COVID-19 pandemic on utilisation of healthcare services: A systematic review. BMJ Open.

[22] Kaufman H.W., Chen Z., Niles J., Fesko Y. (2020). Changes in the Number of US Patients with Newly Identified Cancer Before and During the Coronavirus Disease 2019 (COVID-19) Pandemic. JAMA Netw. Open.

[23] Kempter F., Heye T., Vosshenrich J., Ceresa B., Jäschke D. (2024). Trends in CT examination utilization in the emergency department during and after the COVID-19 pandemic. BMC Med. Imaging.

[24] Wang H., Yang M., Xiong W., Wang Q., Zheng B., Bai Y., Zou K., Li J., Ren J., Chen W. (2024). Noteworthy impacts of COVID-19 pandemic on cancer screening: A systematic review. Fundam. Res..

[25] Coskun M., Cilengir A.H., Cetinoglu K., Horoz M., Sinci A., Demircan B., Uluc E., Gelal F. (2023). Did radiation exposure increase with chest computed tomography use among different ages during the COVID-19 pandemic? A multi-center study with 42028 chest computed tomography scans. Diagn. Interv. Radiol..

[26] He Z., Liu K., Wu L., Wei Q., Shen Q. (2025). Analysis of screen-detected pulmonary nodules before and after the novel coronavirus epidemic: A multicenter retrospective cohort study. Front. Oncol..

[27] Amara A., Trabelsi S., Hai A., Zaidi S.H.H., Siddiqui F., Alsaeed S. (2024). Equivocating and Deliberating on the Probability of COVID-19 Infection Serving as a Risk Factor for Lung Cancer and Common Molecular Pathways Serving as a Link. Pathogens.

[28] LoPiccolo J., Gusev A., Christiani D.C., Jänne P.A. (2024). Lung cancer in patients who have never smoked–an emerging disease. Nat. Rev. Clin. Oncol..

[29] Khan S., Hatton N., Tough D., Rintoul R.C., Pepper C., Calman L., McDonald F., Harris C., Randle A., Turner M.C. (2023). Lung cancer in never smokers (LCINS): Development of a UK national research strategy. BJC Rep..

[30] Romania: Internal Migration from Rural to Urban Areas 2020. Statista. https://www.statista.com/statistics/1257348/romania-internal-migration-from-rural-to-urban-areas/.

[31] Carr S.R., Akerley W., Cannon-Albright L.A. (2018). Genetic Contribution to Nonsquamous, Non-Small Cell Lung Cancer in Nonsmokers. J. Thorac. Oncol..

[32] Cannon-Albright L.A., Carr S.R., Akerley W. (2019). Population-Based Relative Risks for Lung Cancer Based on Complete Family History of Lung Cancer. J. Thorac. Oncol..

[33] Carr S.R., Akerley W., Hashibe M., Cannon-Albright L.A. (2015). Evidence for a genetical contribution to non-smoking-related lung cancer. Thorax.

[34] Tian Y., Ma R., Zhao W., Wang S., Zhou C., Wu W., Yang B., Xin H., Wang H., Li P. (2025). Comprehensive characterization of early-onset lung cancer, in Chinese young adults. Nat. Commun..

[35] Hu M., Tan J., Liu Z., Li L., Zhang H., Zhao D., Li B., Gao X., Che N., Zhang T. (2022). Comprehensive Comparative Molecular Characterization of Young and Old Lung Cancer Patients. Front. Oncol..

[36] Myers D.J., Wallen J.M. (2025). Lung Adenocarcinoma. StatPearls.

[37] Kava C.M., Siegel D.A., Sabatino S.A., Qin J., Henley S.J. (2025). Differences in Lung Cancer Death Rates by Rural vs. Urban Status in Comparison to All-Cancer Death Rates–United States, 1999–2020. Cancer Epidemiol..

[38] Kang H.-R., Kim S.J., Nam J.G., Park Y.S., Lee C.-H. (2024). Impact of Smoking and Chronic Obstructive Pulmonary Disease on All-Cause, Respiratory, and Cardio-Cerebrovascular Mortality. Int. J. Chronic Obstruct. Pulm. Dis..

[39] Nguyen C.V., Le T.T., Nguyen N.H., Hoang K.T. (2023). Socioeconomic inequality in smoking: Evidence from a decomposition analysis. Clin. Epidemiol. Glob. Health.

[40] Chen H., Hu X.-B., Zhou J., He C.-Y., Wang K., Yi Q. (2024). Association of chronic obstructive pulmonary disease with risk of lung cancer in individuals aged 40 years and older: A cross-sectional study based on NHANES 2013–2018. PLoS ONE.

[41] Barnes P.J., Adcock I.M. (2011). Chronic Obstructive Pulmonary Disease and Lung Cancer: A Lethal Association. Am. J. Respir. Crit. Care Med..

[42] Sumiya R., Matsunaga T., Suzuki K. (2025). Lung cancer in young individuals; risk factors and epidemiology. J. Thorac. Dis..

[43] Zhang W., Song X., Song T., Zeng D. (2024). Association between common chronic pulmonary diseases and lung cancer: Mendelian randomization analysis. Discov. Oncol..

[44] Wang I.-J., Liang W.-M., Wu T.-N., Karmaus W.J., Hsu J.-C. (2018). Inhaled corticosteroids may prevent lung cancer in asthma patients. Ann. Thorac. Med..

[45] Lee C.-H., Hyun M.K., Jang E.J., Lee N.R., Kim K., Yim J.-J. (2013). Inhaled corticosteroid use and risks of lung cancer and laryngeal cancer. Respir. Med..

[46] Laguna J.C., Tagliamento M., Lambertini M., Hiznay J., Mezquita L. (2024). Tackling Non–Small Cell Lung Cancer in Young Adults: From Risk Factors and Genetic Susceptibility to Lung Cancer Profile and Outcomes. Am. Soc. Clin. Oncol. Educ. Book.

[47] Zhang Y.-L., Yuan J.-Q., Wang K.-F., Fu X.-H., Han X.-R., Threapleton D., Yang Z.-Y., Mao C., Tang J.-L. (2016). The prevalence of EGFR mutation in patients with non-small cell lung cancer: A systematic review and meta-analysis. Oncotarget.

[48] Melosky B., Kambartel K., Häntschel M., Bennetts M., Nickens D.J., Brinkmann J., Kayser A., Moran M., Cappuzzo F. (2022). Worldwide Prevalence of Epidermal Growth Factor Receptor Mutations in Non-Small Cell Lung Cancer: A Meta-Analysis. Mol. Diagn. Ther..

[49] Mansour M.S.I., Malmros K., Mager U., Lindquist K.E., Hejny K., Holmgren B., Seidal T., Dejmek A., Dobra K., Planck M. (2022). PD-L1 Expression in Non-Small Cell Lung Cancer Specimens: Association with Clinicopathological Factors and Molecular Alterations. Int. J. Mol. Sci..

